# Development and feasibility study of an app (Ladle) for weight loss and behaviour change

**DOI:** 10.7717/peerj.6907

**Published:** 2019-05-15

**Authors:** Jane Ogden, Hazel Maxwell, Adrian Wong

**Affiliations:** 1 School of Psychology, University of Surrey, Guildford, Surrey, United Kingdom; 2 Fittle App Limited (trading as Ladle), London, United Kingdom

**Keywords:** App, Digital, Obesity, Evaluation, Weight loss, Behaviour change

## Abstract

**Background:**

Weight management interventions involving behaviour change often utilise face to face interventions which include evidence based behaviour change strategies yet are costly and time intensive. In contrast, digital interventions cost less and have a wider reach yet tend to lack an evidence base and are less effective.

**Aims:**

The present study therefore aimed to develop an evidence based behaviour change low cost app for weight management and to provide a preliminary analysis of its effectiveness.

**Methods:**

The Ladle app was developed through evidence review and feedback from health care professionals and patients and consists of a 12 week course focusing on six habits and weight loss facilitated through 36 audio psychological lessons and 12 lessons specifically on the six habits. Each lesson was between 2–5 min (approx. 168 min of lessons). It was evaluated in terms of completion rate, weight loss, adoption of the six habits and participant feedback.

**Results:**

The results showed a completion rate of 44%, that 52% of Completers showed weight loss of at least 5%, 79% showed weight loss of at least 3%, the median % weight lost was −5% and the median weight loss was −3.8 kg. Further, by the end of 12 weeks the majority (>80%) of participants had adopted four of the six habits for at least 5 days a week and nearly half (45%) had adopted the remaining two habits for at least 4 days out of 7. Feedback comments were mainly positive (*n* = 80) focusing mostly on the content of the lessons. Some comments were neutral (*n* = 56) and involved a statement of commitment or a description of a challenge and a minority were negative (*n* = 23) describing some technical issues which were addressed as the evaluation progressed.

**Conclusion:**

The new Ladle app offers an evidenced based alternative to more intensive face to face interventions. On preliminary analysis it would seem to have lower completion rates than some more intensive interventions but comparable effectiveness for weight loss. It can also improve habits and is less time-intensive and costly to deliver. Participant feedback was generally positive.

## Introduction

Overweight and obesity have almost tripled worldwide since 1975, and in 2016 39% of all adults were overweight, and 13% were obese (Ng, Fleming & Robinson, 2014; NCD Risk Factor Collaboration, NCD-RisC; WHO, 2015). The highest rates of obesity are found in Tunisia, the USA, Saudi Arabia and Canada, and the lowest are found in China, Mali, Japan, Sweden and Brazil; the UK, Australia and New Zealand are all placed in the middle of the range (WHO, 2015). Overweight and obesity both have psychological and physical health consequences. They are associated with body dissatisfaction, low self esteem, anxiety, low mood and a general lack of confidence (Foresight, 2007; Pereira-Miranda et al., 2017). They also increase the risk of cardiovascular disease, heart attacks, diabetes, joint trauma, back pain, many types of cancer, hypertension and strokes, the likelihood of which increases as a person’s BMI increases greater than 25 (Foresight, 2007; Pereira-Miranda et al., 2017; Mokdad et al., 2004; Romero-Corral et al., 2006; Ortega, Lavie & Sui, 2017). In the UK the prevalence of diabetes increased from 46% to 56% between 1996 and 2005, which can be largely explained by the rise in overweight and obesity (Public Health England, 2015). Obesity is also directly linked with mortality and decreased life expectancy (Mokdad et al., 2004; Romero-Corral et al., 2006; Ortega, Lavie & Sui, 2017; Global BMI Mortality Collaboration, 2016).

Excess body weight also has economic consequences. The global economic cost of obesity is approximately $2.0 trillion, or 2.8 percent of global GDP, paralleling the global impact of smoking or armed violence, war, and terrorism (McKinsey, 2014). Further, the toll of obesity on health-care systems is between 2 and 7 percent of all health-care spending in developed economies which increases to 20% if the cost of treating associated diseases is included (McKinsey, 2014). In the UK, it is estimated that the NHS spent £6.1 billion on overweight and obesity-related ill-health in 2014 to 2015 which is higher than the combined cost of the police, fire service and the judiciary for the same period (Public Health England, 2017).

Obesity and overweight are therefore a health and financial burden. Many people are motivated to lose weight and studies indicate that about 42% of the general population report trying to lose weight in the past year; that about 23% report trying to maintain weight loss in the past year; and about 70% report having ever dieted to lose weight (Santos et al., 2017). Research has therefore evaluated the effectiveness of different weight loss interventions with a focus on face to face and digital approaches.

Face to face weight management programmes can be group interventions or involve one to one support with a health care professional. Both tend to draw upon a wide range of behavioural strategies such as self monitoring, reinforcement, cognitive restructuring, relapse prevention and nutritional information and involve health care expert input. In terms of effectiveness, a review of the evidence by NICE indicated that by one year, those who had received best case evidence based behavioural management from either public sector or private sector weight management behaviour change services such as the NHS, Weight Watchers or Slimming World showed an average weight loss by one year of 2.22 kg (Hartmann-Boyce et al., 2014; Johns et al., 2014). Furthermore, a large scale analysis of weight loss by Slimming World members showed an overall mean change of 3.9 kg and a % weight loss of −4.4% by 12 weeks with those who completed at least 75% of weekly sessions showing a mean weight loss of 6.8 kg and a % weight loss of −7.5% by 12 weeks (Stubbs et al., 2015). Likewise, a trial of either 12 weeks or 52 weeks of Weight Watchers sessions compared to a brief intervention showed mean weight losses by 12 months of −4.75 kg (12 wks group) and −6.76 kg (52 wks group) both of which were greater than the brief intervention (Ahern et al., 2017). Further a trial evaluating Slimming World versus advice illustrated 40% attendance rates at Slimming World, with a mean weight loss of 2.43 kg by one year follow up (Aveyard et al., 2016). Evidence based face to face interventions are therefore moderately effective in producing weight loss, but are costly due to the need for trained professionals to deliver the programme. For example, in the UK, Weight Watchers on referral costs the NHS £45 a participant (Ahern et al., 2011) which, if offered to the 42 million overweight people in the UK would cost the NHS approximately £2 billion. Further an estimate of the incremental cost of Weight Watchers indicated a cost of £159 per kg lost for 52 weeks and £91 per kg lost for 12 weeks (Ahern et al., 2017).

In contrast, digital interventions can be delivered at a lower cost than face to face programmes which is reflected in a recent proliferation of online resources and apps. For example, a scoping review in 2015 identified 393 weight loss apps from 4 commercial app stores (Rivera et al., 2016). Of these, however, only 3 had been scientifically evaluated and only 1 involved health care expert involvement. In addition, the majority were limited in their evidence base. For example, the scoping review concluded that although self-monitoring was the most commonly used approach (35.3%), followed by physical activity support (27.5%) only a quarter used weight assessment (25.4%) or healthy eating support (23.2%) and even less used goal-setting (21.4%), motivational strategies (7.1%), social support (5.3%) or personalized feedback (7/393, 1.8%) (Rivera et al., 2016). In terms of effectiveness, a systematic review and meta -analysis in 2015 identified 12 research studies evaluating apps for weight loss and concluded that the apps were more effective than control groups resulting in a mean of −1.04 kg greater weight loss (Flores Mateo et al., 2015). These apps are therefore less effective than evidence based face to face programmes (Hartmann-Boyce et al., 2014; Johns et al., 2014; Stubbs et al., 2015; Ahern et al., 2017; Aveyard et al., 2016) yet cost less and can be made available to a wider population.

One compromise approach is to provide an app together with human coaching to deliver behavioural strategies. This has been established in the US with the Noom app (Kim, Ray & Veluscek, 2017; Toro-Ramos et al., 2017; Chin et al., 2016; Michaelides et al., 2016). Analysis of this approach indicates greater weight loss compared to apps without coaching (−5.2% by one year follow up) but the employment of human coaches increases the cost of the intervention. For example, consumer access to the Noom course costs $59 (∼£46) per month (Noom, 2018) which is more expensive than consumer access to a Weight Watchers face to face programme where costs start at £17.95 per month (Ahern et al., 2011). In a similar vein, Little et al. (2016) evaluated the impact of combining an internet based intervention with nurse support which was provided either face to face or remotely by email and telephone compared to a control group who received dietician advice and a dietician follow up session. The results showed that both internet groups reported greater weight loss than the control group (−2.64 kg by 12 months) and that whereas those who received internet plus face to face nurse support reported a mean weight loss of −4.14 kg by 12 months those who received internet plus remote nurse support reported a mean weight loss of −3.94.

In summary, the obesity epidemic highlights the need for effective interventions that can reach a wide population. Whilst face to face interventions show some effectiveness they are expensive and limited in their reach. In contrast, apps are less effective but cost less and have wider reach unless human coaching is added which drives the cost up. Some of the key comparison interventions are shown in Table 1 although definitions, timing of interventions and outcomes measures vary between studies so any comparison is tentative.

**Table 1:** Preliminary comparisons of the Ladle app: completion rates and weight loss.

**Type**	**Provider**	**Duration of intervention**	**Completion rate**	**Definition of completer**	**% completer lose ≥ 3%**	**% completers lose ≥ 5%**	**Median kg lost by completer**	**Mean kg lost by completer**	**Median % weight loss completer**	**REF**
Face 2 face	Weight Watchers	12 wks	54%	Attend all 12 weekly sessions	–	57%	−5.4	–	−5.6%	Ahern et al. (2011)
Face 2 face	Counter Weight	12 mths	51%	Not defined	–	43%	–	–	–	Laws et al. (2004)
Face 2 face	Rosemary Conley	12 wks	*Not inferior to WW*	Not defined	*Not inferior to WW*	*Not inferior to WW*	*Not inferior to WW*	*Not inferior to WW*	*Not inferior to WW*	Madigan et al. (2014)
Face 2 face	Slimming World	12 wks	*Not inferior to WW*	Not defined	*Not inferior to WW*	*Not inferior to WW*	*Not inferior to WW*	*Not inferior to WW*	*Not inferior to WW*	Madigan et al. (2014)
Face 2 face	NHS	12 wks	*Inferior to commercial programmes*	Not defined	*Inferior to commercial programmes*	*Inferior to commercial programmes*	*Inferior to commercial programmes*	*Inferior to commercial programmes*		Madigan et al. (2014)
Face 2 face	NHS	12 mths	72%	At least 1/2 of course (4 or more appointments)	–	29%	–	−4.02	–	Logue et al. (2014)
Face to face	Slimming World	12 wks	35%	75% of weekly sessions	–	–	–	−6.8	−7.5%	Stubbs et al. (2015)
Face to face	Slimming World	12 wks	40%	Attendance	–	25% (ITT) (by 12 months)	–	−2.9 (3 mth) ITT -2.43 (12 mth) ITT	–	Aveyard et al. (2016)
Face to face	Weight Watchers	12 wks 52 wks	88.6% 91.6%	Attendance	–	42% (ITT) 57% (ITT) (12 month)	–	−4.84 (ITT) −4.62 (ITT) (3 mth)	–	Ahern et al. (2017)
Internet with nurse support (FtoF OR remote)	NHS	6 months	75%	3 core sessions	–	FtoF 28% (12mnths) Remote 32% (12 months)	–	FtoF −4.14 (12mths) ITT Remote −3.94 (12 mths) ITT		Little et al. (2016)
Digital with coach	The Habit app with councillor lead FB group	16 wks	59%	Used app at least once	–	–	–	–3.33	–	Pagoto et al. (2018)
Digital with coach	Twitter- Behavioral Weight-Loss	12 wks	–	–	–	–	–	−2.4	–	Pagoto et al. (2015)
Digital with coach	Noom	16 wks	84%	Read at least one article per week during any 9 of the 16 weeks. (8% of the course)	–	64%	-6	–	−7.5%	Michaelides et al. (2016)
Digital with coach	The Track	6 mths	96%	Attended 6 month assessment	–	43%	–	−4.4	–	Allen et al. (2013)
Digital no coach	Ladle	12 wks	44%	At least 1/3 of course (or 20 lessons)	79%	52%	−3.5	−3.8	−5.0%	Ladle trial
Digital no coach	MyFitness Pal	12 wks	–	–	–	0%	–0.06	–	0.0%	Laing et al. (2013)
Digital no coach	Tweets, Apps, and Pods	6 mths	90%	Attended 6 month assessment	–	–	–	−2.8	−2.7%	Turner-McGrievy & Tate (2011)
Digital no coach	A mobile health intervention for weight management among young adults	12 wks	–	–	–	–	–	−1.6	–	Hebden et al. (2014)

**Notes.**

ITT, Intention to treat analysis

There is therefore a need for a low cost digital intervention that can be made available to many but is as effective as a face to face programme. The aim of the present study was therefore to develop and evaluate an evidence based app that provides many of the evidence based behaviour change strategies contained within the face to face approaches, involves health care experts in its development but that is also low cost and available for general use by a wider population. This paper describes the development of the new Ladle app and its preliminary evaluation.

## Development of the app

The app development team involved a Professor in Health Psychology with 30 years experience working in eating behaviour and weight management (JO), a registered dietician (SG), two psychology researchers who helped with scripting the habits and lessons (CW and DM), a trained voice artist (RMJ) who delivered the habits and lessons for the audio recordings and the two app developers who run the app company, invested in the app and project managed the whole process (HM, AW).

The process of developing the app followed lean start-up methodology (Ries, 2011). In line with this, the development team worked with clinicians and prospective end users of the app throughout the development process to ensure the Ladle app was developed to meet their needs. The steps of the app development were as follows:

**Step 1: Needs assessment with health care professionals:** Interviews were held with academic researchers specialising in obesity (*n* = 5), Tier 2 and Tier 3 Doctors specialising in obesity (*n* = 3), Consultant Clinical Psychologists (*n* = 2), Dieticians (*n* = 4), Clinical and Programme Leads for Digital Psychological Therapy (both NHS provision and private companies) (*n* = 4), and Clinical Commissioners for Diabetes (*n* = 5) to explore whether a digital, evidenced based, behaviour change app for weight management would be valuable. All those interviewed gave positive feedback.

**Step 2: Needs assessment with overweight and obese patients:** A Google advert describing the proposed Ladle app was set up which drove traffic to an online landing page where a form could be filled out to learn more once the app is ready. Results were 2.6% un-optimised CTR (Click Through Rate) and 6.3% conversion to EarlyBird product. This outperforms the average CTR on all Google Paid Ads of ∼2%, and the average conversion rate for Health and Medical products of 3.27% (WordStream, 2018) indicating that a digital, evidenced based, behaviour change app for weight management would be desirable to end users.

**Step 3: Evidence review:** The structure and content of the app was developed in line with three bodies of research evidence (see 25 for a review): (i) research exploring the predictors of weight loss and weight loss maintenance (Hartmann-Boyce et al., 2014; Johns et al., 2014; Dombrowski et al., 2014; Stubbs et al., 2011; Teixeira et al., 2015; Thomas et al., 2014); (ii) research assessing differences between those who lose weight and maintain this weight loss and those who do not using the National Weight Control Registry together with other research (Thomas et al., 2014; Wing et al., 2008; Wing & Phelan, 2005; Ogden, 2000; Elfhag & Rössner, 2005; Butryn et al., 2007; Epiphaniou & Ogden, 2010a; Epiphaniou & Ogden, 2010b); (iii) qualitative research exploring the characteristics of successful weight loss maintainers (Ogden & Hills, 2008; Epiphaniou & Ogden, 2010a; Epiphaniou & Ogden, 2010b; Greaves et al., 2017). This research highlights the key role of eating breakfast, planning meals, eating meals rather than snacks, avoiding denial and being more active which are reflected in the six behavioural habits. These habits are also reflected in the NHS Choices structure for weight management (NHS, 2018) although recent research has questioned the beneficial role of breakfast (Sievert et al., 2019). Furthermore, this research illustrates the importance of psychological strategies such as goal setting and planning; self monitoring, managing emotional eating; a cost benefit analysis of eating behaviour; cognitive restructuring; relapse prevention; recognising the meaning of food; peer support; eating mindfully; having a positive body image; positive reframing; reinforcement; recognising a behavioural model of weight gain; developing a new identity; and self compassion which form the basis of the 36 psychological lessons. This evidence review resulted in a draft course curriculum for the app.

**Step 4: Focus groups to assess usefulness of proposed course content:** Focus groups (*n* = 2) were conducted with overweight and obese individuals (*n* = 19) and interviews were carried out with spokespeople from obesity charities and obesity interest groups (*n* = 4) to gain feedback on the draft course curriculum. All feedback was positive and minor amendments were made in line with suggestions.

**Step 5: Producing the habit and psychological lesson content:** The app content was written to reflect the evidence review and focus groups. Two psychology researchers were employed to write the scripts for the app and a registered Dietician who specialises in obesity management wrote the recipe cards and created simple rules to avoid the need for calorie counting. This process was overseen by JO, AW and HM.

**Step 6: Building the app:** Software developers were employed to build the Ladle digital weight management course including all habits and psychological lessons. This process was overseen by AW and HM.

**Step 7: Ongoing user feedback:** Users were asked for their ongoing feedback to inform continuous improvement of the Ladle app. This involved either face to face or virtual meetings as well as written feedback within the app itself (*n* = 159 comments; see results for further details). Changes were made to both the structure and content of the app to reflect this feedback.

### The app

The Ladle app delivers a 12 week course focusing on weight loss and 6 behavioural habits supported by 12 short (2–5 mins) audio lessons focusing on the 6 habits and 36 short (2–5 mins) audio psychological lessons. In total the app contains approximately 168 min of recorded information relating to the habits and psychological lessons. Each week consists of one habit and three psychological lessons and the 6 behavioural habits are introduced across the first 6 weeks and reinforced in the second half of the course.

**Week 1**

**Habit:** Healthy breakfast

**Psychological lessons:** Using ‘reason for wanting to lose weight’ as a mantra to help you make the healthy decision; Understanding impact of healthy eating vs exercise; Disliking unhealthy foods (creating aversion).

**Week 2**

**Habit:** Eating at planned times

**Psychological lessons:** Weight and behaviour (accepting that overweight and obesity is caused by behaviour); Practicing hunger tolerance; Emotional eating (replacement activities to emotionally soothe).

**Week 3**

**Habit:** Healthy lunch

**Psychological lessons:** Finding time (commitment to the course and app); Enjoying fruit and vegetables (finding a ‘gateway’ vegetable); The 20 min rule (wait 20 mins if not feeling full up after a meal).

**Week 4**

**Habit:** Healthy dinner

**Psychological lessons:** Feeling comfortable with smaller portions (re-framing feeling after eating e.g., feeling overfull is uncomfortable); Childhood experiences (how feelings of deprivation in childhood can make you want to overcompensate as an adult with getting the most out of every meal); Dealing with slip-ups (avoiding black and white thinking—getting back on track straight away).

**Week 5**

**Habit:** Healthy snacks and drinks

**Psychological lessons:** Support from family and friends (what to say to family and friends to get them to support you); Dealing with food pushers (how to say no); Arranging your environment (arranging the home and work place to support healthy living).

**Week 6**

**Habit:** Being active

**Psychological lessons:** Feeling positive about being active (re-framing getting red and out of breath to something to feel proud of); Positive body image (having a positive body image will help you look after your body as a valuable resource and not damage it with overeating); Celebrating getting to the halfway point.

**Week 7**

**Habit:** Healthy breakfast

**Psychological lessons:** Meal planning techniques; Shopping skills (avoiding the isles with unhealthy snacks, understanding the supermarket can be a trigger); Overcoming feelings of unfairness (everyone has some unfairness in life, having to monitor food intake is yours and you can do it/the feeling of unfairness of missing out on this chocolate bar is not as much as the unfairness of being unhealthy).

**Week 8**

**Habit:** Eating at planned times

**Psychological lessons:** What to do when you’re not the cook (practical tips for not overeating when someone else is cooking); Distinguishing between food desire and hunger; Dealing with external triggers e.g walking past a cake shop.

**Week 9**

**Habit:** Healthy lunch

**Psychological lessons:** Recognising unhelpful rules (e.g., I mustn’t waste food) and how to overcome them; Rewarding yourself for progress; Visualisation (meditating on the future, healthier, slimmer you and thanking you for getting you there).

**Week 10**

**Habit:** Healthy dinner

**Psychological lessons:** Overcoming excuses (I can treat myself because …); Your new identity (re-enforcing that you are now a person who has healthy habits, this is who you are); Using smaller plates.

**Week 11**

**Habit:** Healthy snacks and drinks

**Psychological lessons:** Assertiveness with those that make you feel bad about controlled eating; Reflecting on the benefits of healthy eating as a mantra to help you make the healthy decision; Mindful eating.

**Week 12**

**Habit:** Being active

**Psychological lessons:** Priming yourself for exercise; Feeling confident during exercise (practical tips on what to wear, having a workout buddy etc.); Celebrating finishing the course.

For each habit and lesson participants listened to an audio recording and then answered questions to consolidate their learning. Some also required participants to make personal commitments to use the psychological tool. Information relating to food intake (ie breakfast, lunch, dinner, snacks) uses ‘fist size’ for portion control rather than calorie counting.

**Expert support:** There was also space for participants to ask questions and comment on any aspect of the course. Responses from a Ladle trained professional were given within 24 h.

**Peer support:** Participants could receive peer support by replying to each other’s comments on the different lessons.

### Evaluating the app

The app was evaluated as follows:

#### Methods

##### Design.

The study used a single arm prospective design with data collected at baseline (time 1) and at the end of each of the 12 weeks (t2-13) to assess retention and completion, weight loss and changes in the behavioural habits. In line with recommendations for a feasibility study a control arm was not included at this early stage of evaluation (Bowen et al., 2009). University of Surrey granted ethical approval to carry out the study within its facilities (Ethical application ref: UEC_2017_067_FHMS).

##### Participants.

The app was made available to participants via online adverts (Adwords, Facebook) and through posters at the University. Inclusion criteria were: aged 18 + years; BMI >25; regular access to the internet. Exclusion criteria were; diagnosed with an eating disorder; pregnant; been advised by a doctor to avoid losing weight. Data was collected over a period of 24 weeks. Participants were only allowed to download the app after reading the participant information sheet and giving written consent as part of the sign up process.

#### Measures

Measures were taken of the following:

**(i) Retention and completion**: retention at each stage of the 12 week course was recorded. Participants were defined as starters if they met the following criteria: completed at least 1 week of the course and signed into the course at least twice in the first month; at least 3 days interval between starting course and any weight submission thereafter. Participants were defined as a completer if were defined as a starter and had completed at least 1/3 of the course. These criteria were set at the onset of the study to reflect what was considered a reasonable definition for a new app.

**(ii) Weight loss:** Weight loss was recorded by patient self report through the app throughout the 12 week course. The weight loss goal of ≥ 5% was set as research indicates an association with clinically significant benefits in terms of diabetes and a reduction in cardiovascular risk factors (Wing et al., 1987; Aucott, 2008; Blackburn, 1995).

**(iii) Behavioural habits:** Participants rated 6 behavioural habits from the week that this habit was introduced as a goal. The habits were: Healthy breakfast; Eating at Planned Times; Healthy Lunch; Healthy Dinner; Healthy Snack and Drinks; Being Active. Participants were asked to rate ‘How many days over the past week have you [carried out the habit]’ on a scale ranging from 1 day to 7 days. The behavioural habit goal was set at carrying out the habit at least 5 out of 7 days.

**(iv) Participant feedback:** Participants also gave free text written feedback throughout the course.

### Data analysis

The data were analysed to assess retention and completion rates across the 12 week course, weight loss and adoption of the six behavioural habits. In addition, participant free text feedback was classified into types. Data was analysed using Excel and SPSS and descriptive statistics of distribution. No procedure was used to manage missing values. Missing data were not included in the analysis. No inferential statistics were used so a power calculation was not deemed appropriate.

## Results

### (i) Retention and completion

**Starters:** Eighty-three participants were initially deemed to be starters. However, one participant voluntarily withdrew from the trial and seven participants were excluded from the analysis as their weight submissions were unrealistic on unusable (e.g., stating weight loss of 100 kg in week 1; writing ‘no scales’). Accordingly the final sample consisted of 75 starters. Of these *n* = 63 (84%) were women and *n* = 12 (16%) were men. The mean age was 47 yrs SD 12 (range 19–72 yrs) and the mean BMI at baseline was 31 (SD= 6; range 25-51). Data on ethnic group ( *n* = 45) and post code (*n* = 30) was also collected. From this sample 40 were White; three were Asian, one described themselves as African and one described themselves as other. Further, *n* = 8 were from deprived areas.

**Completers:** Of the 75 starters, *n* = 33 (44%) completed the course and *n* = 42 (56%) dropped out before 4 weeks. The completion rate was therefore 44%. Of these completers 29 were women; four were men; their mean BMI at the start of the course was 31 (*SD* = 5; range 25–50). For those completers with available data for ethnic group (*n* = 18), one was Asian and 17 were White and for those with post code data (*n* = 9) five were from deprived areas.

Overall, the number of people completing each of the 12 weeks was as follows: Wk 1: 100%; Wk 2: 80%; Wk 3: 62%; Wk 4: 44%; Wk 5: 28%; Wk 6: 25%; Wk 7: 22%; Wk 8: 16%; Wk 9: 13%; Wk 10; 12% Wk 11: 12%; Wk 12: 9%

### (ii) Weight loss

The results showed that of the starters (*n* = 75), 24% (*n* = 18) and of the completers (*n* = 33), 52% (*n* = 17) showed weight loss ≥5%. Using a less stringent criteria of ≥3%, 44% (*n* = 33) of the starters and 79% (*n* = 26) of the completers met this criteria. Weight lost in terms of kg and % weight lost for starters (*n* = 75) and completers (*n* = 33) is shown in Table 2.

**Table 2:** Weight loss for starters and completers.

**Weight loss**	**Starters (*n* = 75)**	**Completers (*n* = 33)**
**Weight loss (kg)**		
Mean	−2.38	−3.81
SD	2.55	2.55
Median	−2.0	−3.5
Range	−12 ± 2	−12- +1
Percentile	25 = − 4.050 = − 2.075 = − 0.6	25 = − 4.650 = − 3.575 = − 2.27
**% Weight loss**		
Mean	−2.87	−4.65
SD	3.06	2.74
Median	−2.2	−5.0
Range	−14.29- _2.15	−12.37 ±1.38
Percentile	25 = − 4.9450 = − 2.275 = 0.72	25 = − 5.4750 = − 5.075 = − 3.1

Some participants, however, showed weight gain. Of the starters (*n* = 75) four gained weight (0.19%; 0.53%; 1.05%; 2.15%) and of the completers, one gained weight (1.38%). 13 (17.3%) starters and one (3%) finisher showed no weight change.

### (iii) Behavioural habits

By the end of the course the results showed that of the completers (*n* = 33) a large majority had adopted four of the six behavioural habits for at least 5 days out of 7 as follows: Healthy breakfast: 91% (*n* = 30); Eating at Planned Times: 85% (*n* = 28); Healthy Lunch: 91% (*n* = 30); Healthy Dinner: 91% (*n* = 30). Nearly half had adopted the remaining 2 habits for 4 days out of the 7: Healthy Snack and Drinks: 45% (*n* = 15); Being Active: 45% (*n* = 15).

### (iv) Participant free text feedback

The 75 starters submitted a total of 159 comments. These were classified as either positive (*n* = 80), neutral (*n* = 56) or negative (*n* = 23).

### Positive comments

The positive comments were classified as relating to lesson content (*n* = 56), lesson format (*n* = 1), losing weight (*n* = 5), overall programme (*n* = 8) and expert support (*n* = 10). Positive lesson content comments were spread across all lessons apart from those relating to ‘eating at planned times’ which was deemed particularly useful. Examples of comments are as follows:

**Lesson content:** So happy to have been motivated to look at weight loss differently. I’ve been dieting for over 30 years. The fist for measuring is so simple and brilliant’; ’I like the idea of giving the foods I wish to resist an unappealing name’.

**Lesson format:** ‘I like the audio and then quiz to check I understood and remembered’.

**Losing weight:** ‘So happy to have lost nearly half a stone, I definitely feel thinner and I am getting back into some of my old clothes again’; ‘Still going well another half pound lost this week, despite having a week off work and out of the normal routine’.

**Overall programme:** I’m really enjoying the course so far - early days I know, but it’s making sense’; ‘The course is working well for me at the moment’.

**Expert support:** Thank you Adrian and team, that’s clearer’.

### Neutral comments

The neutral comments were classified as relating to statements of commitment or circumstances (*n* = 22); finding something challenging or expressing a setback (*n* = 19), questions about the course (*n* = 10) or general other (*n* = 5). Of the challenges or setbacks most related either to eating at planned times in terms of the difficulty in having regular meals (*n* = 7) and snacking (*n* = 7). Examples of comments are given below:

**Commitment/statement of circumstances:** ‘Well started today with a healthy breakfast... fist size malted wheats and fist size amount of skimmed milk. Plus my cup of tea, no sugar and a teaspoon of skimmed milk’; ‘I will use the app at 8 every night, so ready to start a fresh the next day’.

**Finding something challenging or expressing a setback: ‘**I am finding it difficult not to snack when I am fed up’; ‘Whoops, I had a setback last night!! - I had an early evening meal -5pm- come 8:30pm I was starving and went off plan!!!!’

**Questions: ‘**What about protein for breakfast such as eggs?’

### Negative comments

The negative comments were classified as technical issues (*n* = 10); the content of the course (*n* = 10) and failure to lose weight (*n* = 3). Of the technical issues most were about navigation and lesson completion (*n* = 6) and of the content of the course most were about exercise being impractical due to injury or disease (*n* = 5). Examples of negative comments are given below:

**Technical issues:** ‘I too have had issues with the programme, I have had to repeat from week 3 in order to get to where I was, so you will have double submissions from me’; ‘’Not relating to this lesson, but I have did have the same issue again with being put back to previous lessons’; ‘Hi, I can’t see the play button for the audio lesson? I can see the quiz but not anything to play before? Thanks’.

**Content:** ‘Due to a chronic back I can’t exercise’; ‘I’m registered disabled; have a chronic back pain chronic fatigue so I’d love to be physically active but sadly can’t physically do it’; ‘Because I have issues with bowels, I cannot eat anything high in fibre, whole grain and struggle with Dairy’.

**Failure to lose weight:** ‘Not found much to change yet. See how it goes’.

## Discussion

Obesity and overweight have physical and psychological health consequences (Foresight, 2007; Pereira-Miranda et al., 2017; Mokdad et al., 2004; Romero-Corral et al., 2006; Ortega, Lavie & Sui, 2017). They also have economic implications for the health care system (McKinsey, 2014). Existing behaviour change programmes either use face to face interventions which are moderately effective, but expensive and have poor reach or digital interventions which have wider reach, cost less but have limited if any effectiveness which is only improved with the availability of a human coach (e.g., (Hartmann-Boyce et al., 2014; Johns et al., 2014; Stubbs et al., 2015; Ahern et al., 2017; Aveyard et al., 2016; Rivera et al., 2016; Chin et al., 2016; Little et al., 2016). The present study therefore aimed to develop and evaluate a digital app based weight loss programme which incorporated the evidence based behaviour change strategies used by face to face interventions but was lower cost and available to the wider population.

The resulting Ladle app was developed using seven steps including expert, health care practitioner and service user input and evidence review. It involved a focus on 6 behavioural habits supported by 36 audio psychological lessons and took 12 weeks to complete. The app was evaluated in terms of completion rates, weight loss, behavioural habits and participant feedback.

The results from the evaluation showed that 75 people were considered starters and of these 44% (*n* = 33) were considered completers. This is lower than some of the studies reporting data for the completion rates for the commercial groups such as Weight Watchers and Slimming World and NHS groups who offer face to face group sessions (e.g., Ahern et al., 2011; Laws R; Counterweight Project Team, 2004; Logue et al., 2014). It is also lower than available apps that offer a human coach (e.g., Pagoto et al., 2018; Allen et al., 2013; Michaelides et al., 2016). It is, however, comparable or higher than a few of these more intensive interventions (Stubbs et al., 2015; Aveyard et al., 2016). No completion rate data is available for apps without a human coach. This suggests that although a digital app with no coach can have wide reach and attract a high number of users fewer people may complete the course than for a more intensive face to face weight management programme.

In terms of actual weight loss, the results were much more positive. In particular, 52% showed at least 5% weight loss which has been associated with improved health status (Wing et al., 1987; Aucott, 2008) which was comparable or higher than that reported by some face to face interventions (e.g., Ahern et al., 2011; Ahern et al., 2017; Laws R; Counterweight Project Team, 2004; Logue et al., 2014; Aveyard et al., 2016). It is also higher than some digital apps with a coach (Michaelides et al., 2016; Allen et al., 2013). In addition, 72% showed greater than 3% weight loss which also brings benefits, not only in terms of health but also as an incentive to persist with weight loss efforts. Furthermore, in terms of absolute weight loss (mean −3.8 kg; median −3.5 kg) or percentage weight loss (mean 4.65%; median −5%) Ladle was comparable to available data for several face to face interventions (e.g., Aveyard et al., 2016; Logue et al., 2014; Ahern et al., 2011) and an improvement on apps with no human coach (e.g., Turner-McGrievy & Tate, 2011; Laing et al., 2014).

The Ladle app was also evaluated in terms of 6 behavioural habits, namely healthy breakfast, lunch, dinner, healthy snacks and drinks, eating at planned times and being active which have been consistently linked with weight loss (Ogden, 2018; NHS, 2018). The results showed that by the end of the 12 week course the large majority of participants had adopted 4 of the 6 habits and nearly half had adopted the remaining two habits which should not only bring about immediate health benefits but set the scene for longer term behaviour change and weight loss in the future.

Finally the app was evaluated in terms of participant feedback which was mostly positive. In particular, participants were extremely positive about the content of the lessons. Their neutral comments described statements of commitment and any challenges they faced with adhering to the programme and the negative comments mostly reflected technical issues which were addressed throughout the evaluation. The app was therefore found to be acceptable to this patient group.

There are some problems with the current study that need to be considered. First the study utilised a single arm prospective design without randomisation and without a control group. This was due to the desire to collect preliminary data regarding completion rates and weight loss as a first stage to testing the app. Second, weight loss data was collected from within the app and was therefore self report which may reflect issues of social desirability and error. Further, comparisons with existing studies are problematic due to the vast variation in designs used, interventions, duration of interventions, definitions of completions rates and weight loss and whether the data were collected as part of a larger randomised control trial or a service evaluation. These comparisons should therefore be seen as tentative and a preliminary analysis. This present study, however, provides a detailed insight into the process of developing an evidence based app and some preliminary insights into its usefulness and impact on weight loss and habit change.

## Conclusion

In summary, this feasibility study aimed to develop and evaluate a weight loss app that was low cost with a wide reach but based upon the evidence and utilising the evidence based behaviour change strategies associated with sustained weight loss maintenance. The Ladle app offers a 12 week course designed to promote 6 key habits and weight loss facilitated by psychological lessons. The results of the analysis indicate that although completion rates were lower than many face to face interventions and apps with a human coach, weight loss was comparable to several of these more intensive and costly forms of weight management and better than that that achieved by other apps with no human coach. This new app therefore offers an evidence based digital approach to weight management which is as effective as other approaches but can be delivered at lower cost to a wider population.

##  Supplemental Information

10.7717/peerj.6907/supp-1 Data S1 Ladle raw data Data from the trial for starters and finishers.

10.7717/peerj.6907/supp-2 File S1 Accessing the Ladle app

## Additional Information and Declarations

### Data Availability

The following information was supplied regarding data availability: Raw data from the study is available in Data S1.
