# Supplementary material for: Development and feasibility study of an app (Ladle) for weight loss and behaviour change
Source: PeerJ. 2019 May 15;7:e6907. doi: 10.7717/peerj.6907 (PMC6525583; doi:10.7717/peerj.6907)
Supplement: File S1 [file peerj-07-6907-s002.docx]

You can access the Ladle App as if you were a participant by using this log on:

URL: https://ladle.teachable.com/

Username: demotheladleapp@gmail.com

Password: APPLETREE

**Please note:** We have to give you a participant account. We cannot give you an ‘admin account’ as you would then be able to see identifiable participant characteristics, and be able to edit the app.
